# Detection of the “Crossed Aorta Sign” during Echocardiography before Angiography

**DOI:** 10.1155/2017/9249821

**Published:** 2017-12-14

**Authors:** Laura Massobrio, Alberto Valbusa, Giovanni Bertero, Fabrizio Montecucco, Gian Marco Rosa, Gian Paolo Bezante, Claudio Brunelli

**Affiliations:** ^1^Division of Cardiology, Department of Internal Medicine, University of Genoa, 6 Viale Benedetto XV, 16132 Genoa, Italy; ^2^Division of Cardiology, IRCCS AOU San Martino-IST, 10 Largo Benzi, 16132 Genoa, Italy; ^3^First Clinic of Internal Medicine, Department of Internal Medicine, University of Genoa, 6 Viale Benedetto XV, 16132 Genoa, Italy; ^4^Ospedale Policlinico San Martino, 10 Largo Benzi, 16132 Genoa, Italy; ^5^Centre of Excellence for Biomedical Research (CEBR), University of Genoa, 9 Viale Benedetto XV, 16132 Genoa, Italy

## Abstract

We report the case of an anomalous circumflex (Cx) origin from the right sinus of Valsalva with retroaortic course observed in a modified apical four-chamber view during transthoracic study (TTE). This finding is known as the “crossed aorta sign.” Usually, the diagnosis of this congenital anomaly of coronary circulation is established during coronary angiography. In this case, the diagnosis was performed by echocardiography before angiography. We believe that recent improvements in echocardiography increase the potential of this imaging technology also in the diagnosis of coronary artery anomalies.

## 1. Introduction

The coronary artery anomalies can be classified as abnormalities of origin, course, and termination [1]. These congenital anomalies can affect all coronary arteries. In adult patients, the prognosis of coronary artery anomalies is mainly determined by the arterial course to aorta and pulmonary arteries and may be associated with clinical scenarios, such as congestive heart failure, arrhythmia, myocardial infarction, syncope, and sudden death [2]. Here, we reported a case of coronary anatomic variation of the left circumflex artery originating from the right sinus of Valsalva.

## 2. Case Report

**Figure 1:**
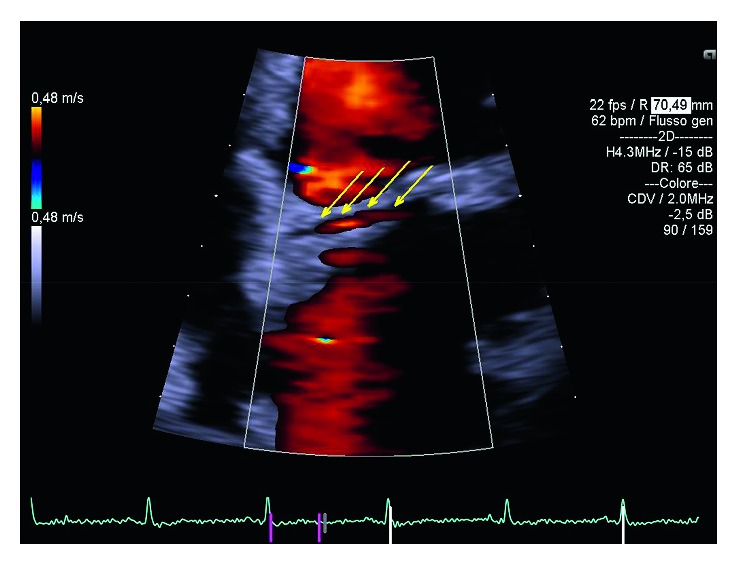
Presentation of anomalous circumflex course. Arrows and color-Doppler indicate its course.

**Figure 2:**
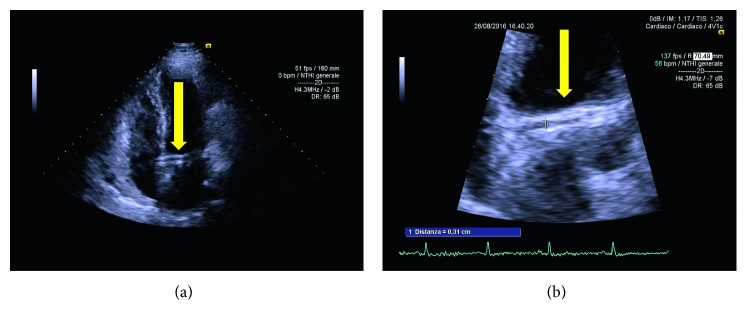
(a-b) Presentation of retroaortic course of circumflex coronary, suggesting “crossed aorta sign” in a modified apical four-chamber view behind left atrium during transthoracic study (arrows).

**Figure 3:**
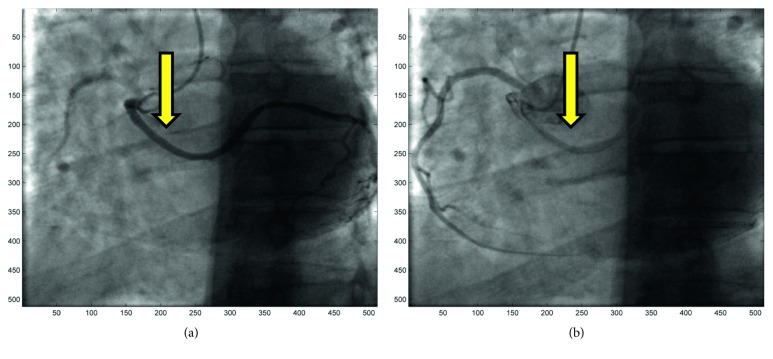
(a-b) Angiographic view of the anomalous circumflex origin from right Valsava sinus (arrows).

We report the case of a 45-year-old Caucasian man with a medical history of hypertension and dyslipidemia and a family history of ischemic heart disease, who was admitted to our acute coronary care unit with diagnosis of acute coronary syndrome (ACS). The patient has given his informed consent for participation in the research study. The twelve-lead electrocardiogram showed sinus rhythm, widespread T-wave inversion in anterior-lateral leads. The transthoracic echocardiogram (TTE) revealed a normal left ventricle (LV) ejection fraction (55%), with limited apical wall motion abnormality. Furthermore, on the basis of a “crossed aorta sign,” we suspected the anomalous circumflex (Cx) coronary artery with probable origin from the right sinus of Valsalva (Figure 1) [3]. In a five-chamber apical view, Cx seemed to cross the aorta perpendicularly to aortic long axis, suggesting retroaortic course (Figures 2(a) and 2(b)), as previously described by Wierzbowska and colleagues. The authors also described the “bleb sign”: the cross section of retroaortically coursing Cx, forming a particular sign in mitroaortic angle in the transoesophageal echocardiography (TEE) long-axis aortic view. This is a new type of echocardiographic sign, which can help to detect the retroaortic coursing of Cx. In our case, the use of the TEE technic was not necessary, since the abnormal course of the Cx was already well detectable by transthoracic echocardiography [3]. During angiography, the anomalous Cx origin from the right sinus was confirmed (Figures 3(a) and 3(b)). Anyway, the patient underwent successful stenting of the anterior descending artery, the culprit vessel. The coronary artery anomalies are classified into abnormalities of origin, distribution, and termination. The anomalous left Cx may arise from a separate ostium within the right sinus, or very unusually as a proximal branch of the right coronary artery with the approximate incidence of 0.37 to 0.7% in all patients [2, 4].

## 3. Discussion

Congenital anomalies of coronary arteries are detected in about 1% in patients undergoing coronary angiography. In the specific case of the anomalous left circumflex artery, it may originate from a separate ostium within the right sinus, or as a proximal branch of the right coronary artery with the approximate incidence of 0.37 to 0.7% in all patients [2, 4]. In these cases, it usually courses inferiorly and posteriorly to the aorta to enter the left atrioventricular groove. Usually, the diagnosis of this congenital anomaly of coronary circulation is established during coronary angiography, and stent implantation generally facilitates an echocardiographic view of the vessel course. In our case, the diagnosis was performed by echocardiography, before angiography, detecting “crossed aorta sign” [3], a novel echocardiographic sign, in an apical five-chamber view in which Cx seemed to cross the aorta perpendicularly to aortic long axis. At echocardiographic exam, the “crossed aorta sign” can be confused with the course of the coronary sinus. However, considering the coronary anatomy, the coronary sinus course is usually posterior to the anomalous circumflex course so that the coronary sinus is more easily visualized through the four-chamber apical view, while “crossed aorta sign” can be showed better in the five-chamber apical view, or in a modified four-chamber apical view. In addition, a feature that differentiates the two echographic anatomical structures is the thickness of the vessel wall: the circumflex has thicker walls than the coronary sinus. In most cases, the anomalous origin of Cx is a benign anomaly. Its detection by transthoracic echocardiogram can help cardiologists to prevent clinical complications. In adult patients, the prognosis of coronary artery anomalies, considering a higher vulnerability to atherosclerosis, is mainly determined by the relationship between arterial pathways, aorta, and pulmonary arteries. These coronary anomalies may be associated with congestive heart failure, arrhythmia, myocardial infarction, syncope, and sudden death [2].

## 4. Conclusion

Usually, the diagnosis of this congenital anomaly of coronary circulation is established during coronary angiography, and stent implantation generally facilitates an echocardiographic view of the vessel course. Usually, the coronary stents facilitate the visualization of the vessel course, allowing the observation of a novel echocardiographic sign in an apical five-chamber view. This is due to the fact that implanted stents make the anomalous Cx hyperechogenic and easier to be noticed at echocardiography. Our case is particular, since Cx coursing detection was found before stent implantation [3]. In our case, the diagnosis was performed by echocardiography, before angiography, detecting “crossed aorta sign” [3], a novel echocardiographic sign, in an apical five-chamber view in which Cx seemed to cross the aorta perpendicularly to aortic long axis. We believe that the development of echocardiographic technologies will increase the potential role of echo also in the diagnosis of coronary artery anomalies and other pathological entities that were previously misdiagnosed.
